# Multivalvular Endocarditis With *Granulicatella adiacens* in a 2-Year-Old Boy With Ventricular Septal Defect

**DOI:** 10.1016/j.jaccas.2024.102525

**Published:** 2024-09-18

**Authors:** Christopher Schmitt, Ranjit Philip, Christopher Knott-Craig, Nathan Stecchi, Nithya Swaminathan

**Affiliations:** a Le Bonheur Children’s Hospital, Heart Institute, Memphis, Tennessee, USA; b Division of Pediatric Cardiology, University of Tennessee Health Science Center, Memphis, Tennessee, USA

**Keywords:** Duke criteria, infective endocarditis, ventricular septal defects

## Abstract

A 2-year-old boy with a hemodynamically insignificant ventricular septal defect was found to have polyvalvular endocarditis, eventually requiring replacement of the pulmonary and mitral valves with a pulmonary conduit and a mechanical valve. Cultures grew *Granulicatella adiacens*, listed on the microbiological criterion of the updated Duke International Society for Cardiovascular Infectious Diseases criteria.

## Graphical Abstract

**Figure:**
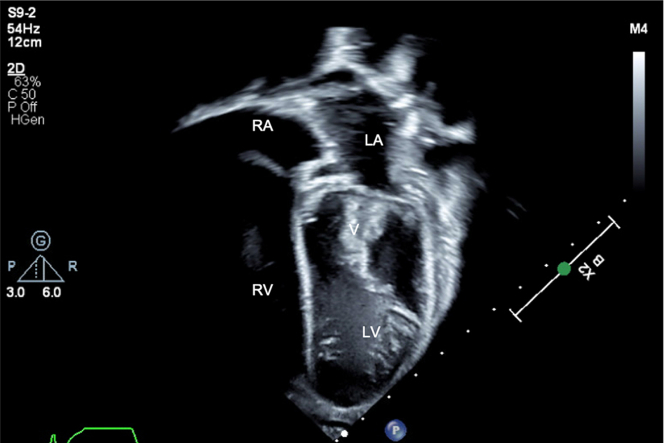

## History of Presentation

A 2-year-old boy was admitted for workup of a fever of unknown origin after almost daily fevers for 2 to 3 weeks. He was diagnosed with otitis media by his primary pediatrician and prescribed a 10-day course of amoxicillin. His fevers resolved for 3 to 5 days before returning. He had no other symptoms other than increased fussiness.

**Learning Objectives**

• To understand that patients with hemodynamically insignificant ventricular septal defects are still at increased risk for infectious endocarditis.
• To know that the 2023 Duke International Society of Cardiovascular Infectious Diseases criteria now include additional common bacterial species (including *Granulicatella*) and how to use of genomic sequencing.

Vital signs showed he was febrile (39.7 °C), tachycardic (148 beats/min), and with normal blood pressure (94/66 mm Hg) and saturations (97%). The physical examination showed irritability; tachycardia; a harsh holosystolic murmur over the left sternal border; a blowing systolic murmur over the apex, radiating to the axilla; an early systolic murmur over the left upper sternal border radiating throughout back and the upper chest; and a diastolic murmur over the left and right upper sternal borders.

## Past Medical History

The patient had a history of prematurity at 35 weeks for maternal preeclampsia; failure to thrive as an infant, treated with increased fortification of feeds; a small, hemodynamically insignificant ventricular septal defect (VSD) that was diagnosed at 1 to 2 months (given the size, it was not believed to be contributory to his failure to thrive and so was medically managed); and iron deficiency anemia, treated with oral iron. There had been no procedures or surgeries in the past 6 months.

## Differential Diagnosis

The differential diagnosis included bacterial endocarditis, myocarditis, acute rheumatic fever, bacteremia, pneumonia, respiratory viral infection, other infectious etiologies, and oncologic diseases.

## Investigations

In the emergency department, blood cultures and laboratory values were drawn. Notable results included leukocytosis (19.0 K/μL; 62% neutrophils), thrombocytopenia (125 K/μL), and elevated inflammatory markers (erythrocyte sedimentation rate = 88 mL/h, C-reactive protein = 67 mg/L, ferritin = 144 ng/mL).

**Figure 1:**
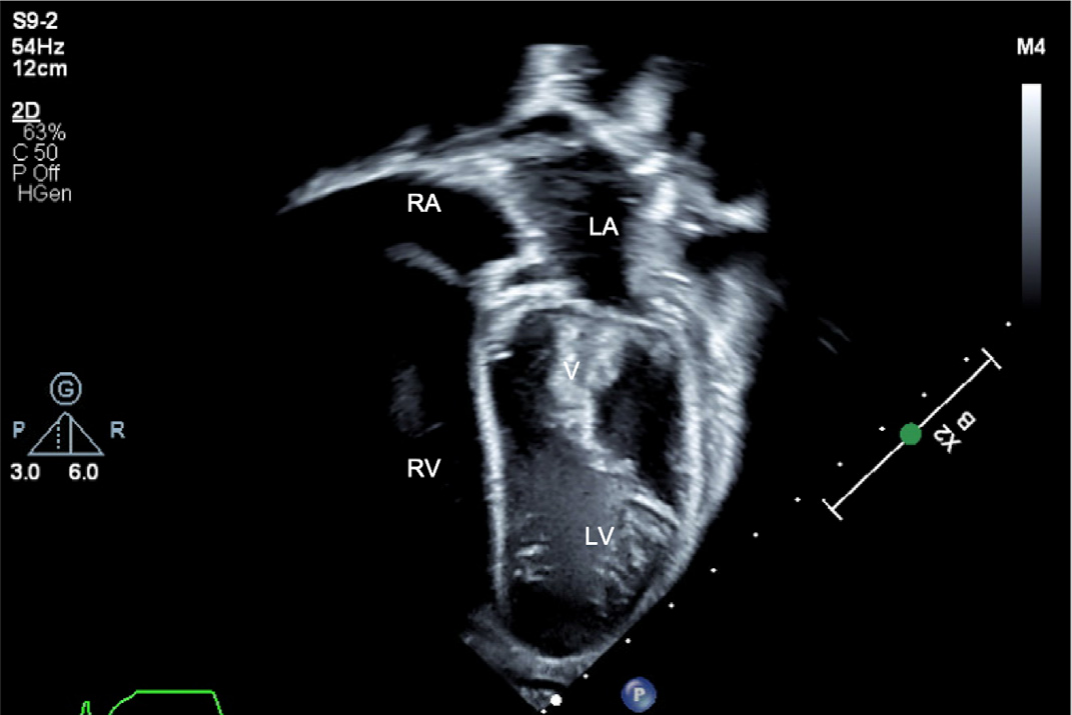
Echocardiogram of Vegetations: Mitral Valve Transthoracic apical 4-chamber view demonstrating a 24 × 10-mm vegetation on the mitral valve. LA = left atrium; LV = left ventricle; RA = right atrium; RV = right ventricle; V = vegetation.

**Figure 2:**
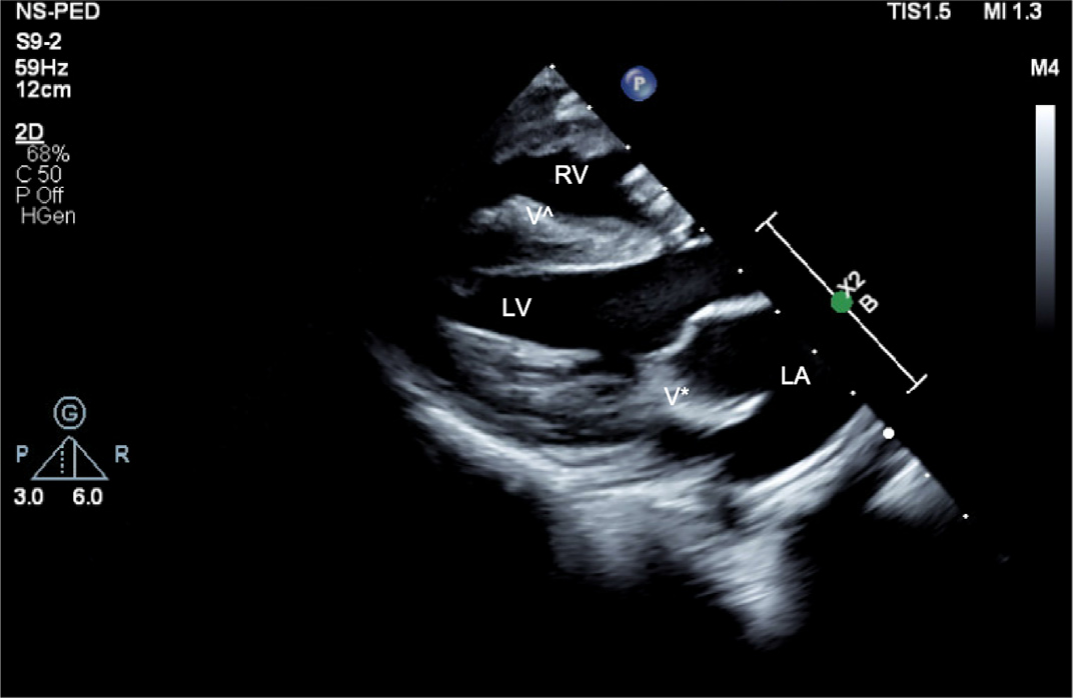
Echocardiogram of Vegetations: Mitral Valve and Right Ventricle Transthoracic parasternal long-axis view demonstrating the 24 × 10-mm vegetation (V∗) on the mitral valve. Another vegetation (Vˆ) is seen in the right ventricular outflow tract adhering to the septum, in proximity to ventricular septal defect. Abbreviations as in Figure 1.

**Figure 3:**
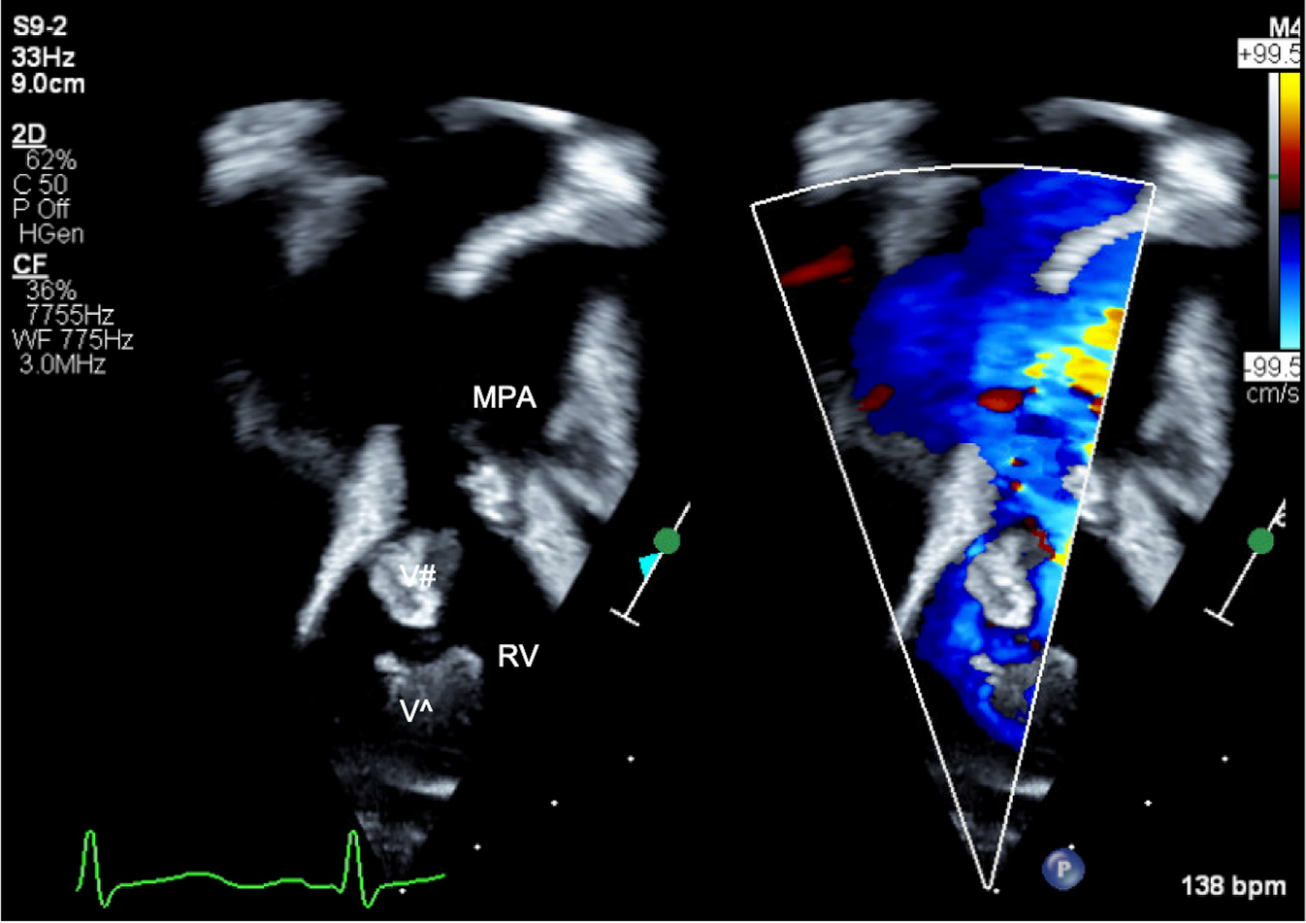
Echocardiogram of Vegetation: Pulmonary Valve Transthoracic apical view focused on the pulmonary valve showing both vegetations on the pulmonary valve (V#) and in the right ventricular outflow tract (Vˆ). MPA = main pulmonary artery; RV = right ventricle.

An echocardiogram showed large echo-bright mobile masses on mitral, pulmonary, and tricuspid valves (Figure 1, Figure 2, Figure 3, Video 1, Video 2, Video 3) measuring 24 × 10 mm (mitral valve), 14 × 10 mm (pulmonary valve), and 10 × 3 mm (tricuspid valve, later confirmed to be adherent to the right ventricular outflow tract wall), which prolapsed in and out of the VSD. There was moderate mitral regurgitation, flow acceleration across the right ventricular outflow tract (peak gradient = 33 mm Hg), mild pulmonary regurgitation, and moderate tricuspid valve regurgitation. The left ventricle was mildly dilated (stable from before), the VSD was small with minimal left to right shunting, and there was normal function. Serial blood cultures all grew *Granulicatella adiacens*.

## Management

**Figure 4:**
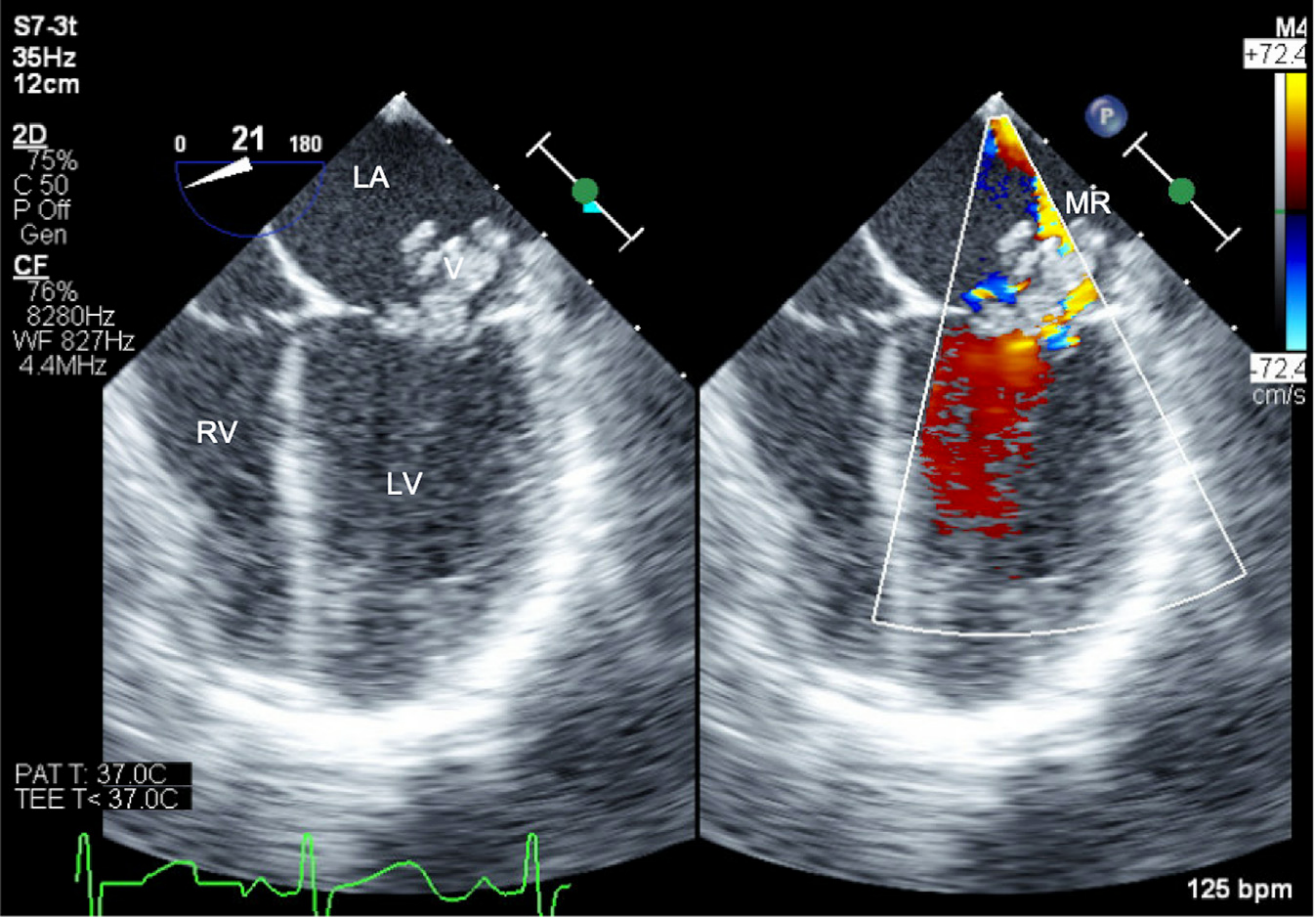
Transesophageal Echocardiogram of Vegetation Four-chamber view demonstrating a large vegetation on the mitral valve, with resultant mitral regurgitation (MR). Abbreviations as in Figure 1.

**Figure 5:**
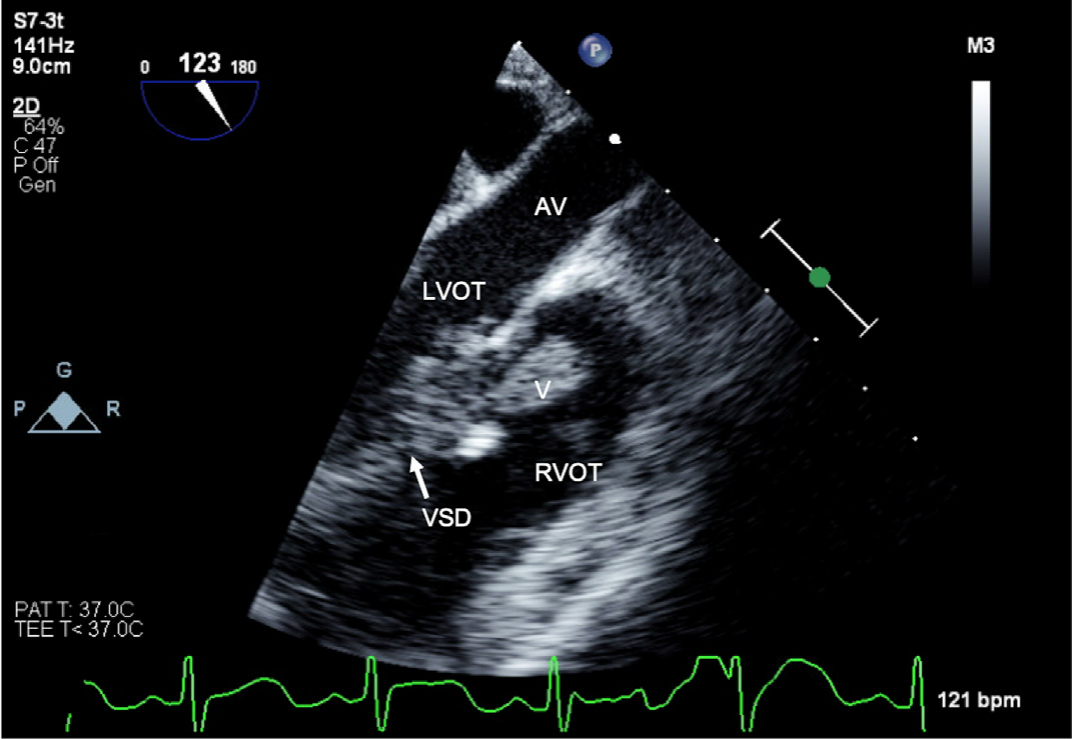
Transesophageal Echocardiogram of Several Vegetations Long-axis view demonstrating a vegetation in the right ventricular outflow tract (RVOT), adjacent to the ventricular septal defect (VSD). AV = aortic valve; LVOT = left ventricular outflow tract.

After the echocardiogram, the patient was presumptively treated for infectious endocarditis (IE) with ceftriaxone and vancomycin. Given the large left sided lesion, he was transferred to the cardiothoracic intensive care unit for frequent neurologic checks. A computed tomography of the brain did not show septic emboli. He was treated with intravenous antibiotics for 48 to 72 hours before surgical intervention. In the operating room, he was found to have massive vegetations of the anterior and posterior leaflets of the mitral valve, of the pulmonary valve, much of the right ventricular outflow tract, and of the VSD itself (Figures 4 and 5, Video 4, Video 5, Video 6).

## Discussion

VSDs are one of the most common congenital heart diseases, with isolated defects occurring in 2 of 6 in 1,000 live births.^1^, ^2^, ^3^ Perimembranous defects constitute 70% to 80% of VSDs and involve the membranous septum; adjacent to the muscular, inlet, and outlet septum; behind the septal leaflet of the tricuspid valve; and below the commissure of the right and noncoronary leaflet of the aortic valve.^2^^,^^4^ Unlike muscular VSDs, only 35% to 40% of perimembranous VSDs spontaneously close, occurring through both muscular septal ingrowth and occlusion from tricuspid valve tissue.^3^^,^^4^ The natural course of unrepaired defects depends on their size and amount of shunting, ranging from clinically asymptomatic to left-sided volume overload, pulmonary hypertension, aortic valve prolapse or regurgitation, infundibular hypertrophy, congestive heart failure, and death.^2^^,^^3^ There is also an increased incidence of IE in unrepaired defects, with studies showing a range of 0.5% to 4% in rates of infection.^3^^,^^4^ Thus, varying opinions have been offered on the optimal approach to hemodynamically insignificant VSDs, such as watchful waiting, surgical closure, and percutaneous closure.^2^^,^^5^

IE is more common in patients with VSDs than in the general population and occurs when there is endocardial injury, exposing underlying collagen and allowing microbes to adhere to the endocardium.^1^^,^^5^^,^^6^ Studies show that defects with restrictive defects and a left ventricle to right atrial shunt were more likely to have IE (8.5% vs 0.3% in restrictive defects without left ventricular to right atrial shunts), which may be caused by the high velocity creating chaotic, turbulent flow, with injury to the tricuspid valve.^7^

Streptococcal species constitute about 50% of endocarditis, of which 5% to 10% are caused by nutritionally variant streptococcus, including species like *G adiacens*.^8^ These nutritionally variant streptococcus species are part of the normal oral flora and in the gastrointestinal and genitourinary tracts. They are fastidious and require growth in specialized medium and so are believed to be responsible for some portion of culture-negative endocarditis. With slower and more insidious courses, IE cases caused by these bacteria have higher rates of septic embolization, heart failure, and death, along with higher rates of treatment failure and relapses.^6^ *Granulicatella* species were added as typical pathogens in the 2023 Duke International Society of Cardiovascular Infectious Diseases criteria, in addition to *Staphylococcus lugdunensis, Enterococcus faecalis*, *Abiotrophia* species, *Gemella* species, and all streptococci except *S pneumoniae* and *S pyogenes*.^9^

## Follow-Up

The patient eventually underwent excision of all vegetations, replacement of the right ventricular outflow tract and pulmonary valve using a 22-mm decellularized pulmonary homograft, patch closure of the VSD, and repair of the tricuspid valve. Because the mitral valve could not be spared, he underwent replacement of the mitral valve using an inverted 19-mm aortic mechanical valve. He was transitioned to ceftriaxone and gentamicin, for 6 and 2 weeks, respectively, which was completed in an outpatient setting.

## Conclusions

This case highlights the importance of keeping IE on the differential diagnosis in patients with small, hemodynamically insignificant VSDs. It demonstrates that as we have improved our understanding of the microbiology of “culture-negative” endocarditis, we should be aware that nutritionally variant streptococcal species can be associated with more adverse outcomes, a more prolonged course, and higher rates of treatment failures and as such have been added as criterion in the 2023 Duke International Society of Cardiovascular Infectious Diseases diagnostic classification.^9^ However, similarly to questions regarding the specificity of the 2000 modifications of the Duke Criteria in the pediatric population, more research needs to be done to evaluate the sensitivity and specificity of the new updates in the pediatric population.^10^

## Funding Support and Author Disclosures

The authors have reported that they have no relationships relevant to the contents of this paper to disclose.

## Appendix

Video 1 Echocardiogram of Endocarditis on the Mitral Valve Transthoracic apical 4-chamber view demonstrating a 24 × 10-mm vegetation (V) on the mitral valve. The left atrium and left ventricle are mildly dilated, and there is normal biventricular function.

Video 2 Echocardiogram of Endocarditis on the Mitral Valve and Right Ventricle Transthoracic parasternal long-axis view demonstrating the 24 × 10-mm vegetation (V∗) on the mitral valve. Another vegetation (Vˆ) can be seen in the right ventricular outflow tract adhering to the ventricular septum, in proximity to the VSD, which can be seen moving between the right ventricle and left ventricle.

Video 3 Echocardiogram of Endocarditis on the Pulmonary Valve Transthoracic parasternal long-axis view focused anteriorly, demonstrating a 14 × 10-mm vegetation (V#) on the pulmonary valve. The mitral valve vegetation (V∗) and vegetation in the right ventricular outflow tract (Vˆ) can also be seen.

Video 4 Transesophageal Echocardiogram of Vegetation on the Mitral Valve Transesophageal 4-chamber view demonstrating the large vegetation on the mitral valve, with resultant mitral regurgitation. The vegetation can be seen prolapsing both in and out of the left atrium and left ventricle.

Video 5 Transesophageal Echocardiogram of Vegetation in the Right Ventricle Transesophageal long-axis view demonstrating left to right shunting through a small perimembranous VSD. Adjacent to the VSD, there is a vegetation in the right ventricular outflow tract.

Video 6 Transesophageal Echocardiogram of Vegetations Transesophageal short-axis view demonstrating the vegetation in the right ventricle outflow tract with resultant flow acceleration. The tricuspid valve does not have a vegetation. There is a small perimembranous VSD with left to right shunting.
